# Study protocol: Care of Late-Stage Parkinsonism (CLaSP): a longitudinal cohort study

**DOI:** 10.1186/s12883-018-1184-3

**Published:** 2018-11-05

**Authors:** Monika Balzer-Geldsetzer, Joaquim Ferreira, Per Odin, Bastiaan R. Bloem, Wassilios G. Meissner, Stefan Lorenzl, Michael Wittenberg, Richard Dodel, Anette Schrag

**Affiliations:** 1 Department of Neurology, Philipps-University Marburg, Marburg, Germany; 2 Instituto de Medicina Molecular Universidad di Lisboa, Lisboa, Portugal; 3 Department of Neurology, Lund University Hospital, Lund, Sweden; 4 Donders Institute for Brain, Cognition and Behaviour, Department of Neurology, Radboud University Medical Center, Nijmegen, The Netherlands; 5 Service de Neurologie, CHU de Bordeaux, 33000 Bordeaux, France; 6 Institut des Maladies Neurodégénératives, University de Bordeaux, UMR 5293, 33000 Bordeaux, France; 7 Interdisziplinäres Zentrum für Palliativmedizin und Klinik für Neurologie Universität München - Klinikum Großhadern, Munich, Germany; 8 Institute of Nursing Science and –Practice, Salzburg, Austria; 9 Coordinating Centre for Clinical Trials (KKS), Philipps-University Marburg, Marburg, Germany; 10 Department of Geriatric Medicine, University Hospital Essen, Essen, Germany; 11 UCL Institute of Neurology, University College London, Royal Free Campus, Rowland Hill street, NW3 2PF, London, UK

**Keywords:** Late stage parkinsonism, Care provision, Non-motor complications, Quality of care, Health-related quality of life, Health-economic evaluation, Patient-reported outcomes

## Abstract

**Background:**

Parkinson’s disease (PD) is a chronic progressive disorder leading to increasing disability. While the symptoms and needs of patients in the early stages of their disease are well characterized, little information is available on patients in the late stage of the disease.

**Methods/design:**

The Care of Late-Stage Parkinsonism (CLaSP) study is a longitudinal, multicenter, prospective cohort study to assess the needs and provision of care for patients with late stage Parkinsonism and their carers in six European countries (UK, France, Germany, Netherlands, Portugal, Sweden). In addition, it will compare the effectiveness of different health and social care systems. Patients with Parkinsonism with Hoehn and Yahr stage ≥IV in the “On”-state or Schwab and England stage 50% or less are evaluated at baseline and three follow-up time-points. Standardised questionnaires and tests are applied for detailed clinical, neuropsychological, behavioural and health-economic assessments. A qualitative study explores the health care needs and experiences of patients and carers, and an interventional sub-study evaluates the impact of specialist recommendations on their outcomes.

**Discussion:**

Through the combined assessment of a range of quantitative measures and qualitative assessments of patients with late stage parkinsonism, this study will provide for the first time comprehensive and in-depth information on the clinical presentation, needs and health care provision in this population in Europe, and lay the foundation for improved outcomes in these patients.

**Trial registration:**

The protocol was registered at ClinicalTrials.gov as NCT02333175 on 07/01/2015.

## Introduction

Parkinson’s disease (PD) is the second most common neurodegenerative disorder, affecting approximately 1.2 million people in Europe [1]. There is increasing disability with disease progression, but whilst there is an abundant number of studies on the health and social care needs of patients in the early stages of PD, there is surprisingly little information on the medical and care needs of the late stage population, and how these needs are currently met. In addition, there is little information on the use and effectiveness of the pharmacological and non-pharmacological interventions for PD in these late stages, when there may be multiple features and comorbidities. These include motor complications and non-motor symptoms such as behavioral and psychological symptoms, autonomic disturbances and sleep disorders, which can contribute to the burden of the disease to patients and their carers [2, 3] and may lead to nursing home placement and higher mortality [3–5]. Most studies on the management and care needs of patients with PD include only small subgroups in the late stages of the disease [6, 7] and patients in Hoehn and Yahr stages IV and V [8] are usually excluded from clinical trials [9]. In specialist practice, the proportion of patients in the late stages is also underrepresented as they are often too disabled to attend hospital or office-based appointments and do not receive adequate care [10]. It is therefore currently relatively unknown what the exact health care needs are in patients with late stage Parkinsonism, and no large study has evaluated the provision of health care for these patients and associated costs across different European health care systems. Small studies point to an increased use of health as well as social care resources (hospitalization and institutionalization) in late PD stages [11–14] and a high need for informal care with increased caregiver burden [12, 14].

The Care of Late-Stage Parkinsonism (CLaSP) study aims to establish a large European cohort of patients with parkinsonism in the late stage of the disease (i.e. HY stages I*V*/V in the “On”-stage or Schwab and England stage 50% or less) in six European countries to examine the health and social care needs of this patient group and their carers, available health care provision and costs to society. Alongside the cohort study, eligible patients are randomised in an interventional trial evaluating the impact of specialist recommendations in patients in late stages of Parkinsonism, and a qualitative study explores the health care needs and experiences of patients and carers. This article describes in detail the methodology of the study and the assessments used in this large and unique cohort. An abstract on the study design has been previously published [15].

## Methods

### Study design

The CLaSP study is a longitudinal multicenter cohort study of patients with late stage Parkinsonism and their carers. In the six European health care systems (London and Luton, UK; Marburg-Giessen, Essen, and Munich, Germany; Nijmegen, The Netherlands; Bordeaux, France; Lisbon, Portugal; and Lund, Sweden) patients with late-stage Parkinsonism and their informal carers are identified from neurology, care of the elderly, palliative care, and primary care settings. Patients for the qualitative interviews and for the intervention trial are recruited from the participants in the cohort study.

### Cohort study

#### Inclusion criteria

Patients are eligible for enrolment in the CLaSP study, if they had been diagnosed for at least seven years with Parkinsonism and are classified as Hoehn and Yahr stage (HY) IV or V in the “On”-state OR have developed significant disability (Schwab and England stage ≤50%) in the “On”-state [16]. Established clinical criteria (UK Parkinson's Disease Society Brain Bank Diagnostic Criteria [17]) are applied to distinguish subjects with PD from those with different atypical parkinsonian syndromes. However, since distinction in late stages of the disease can be difficult and patient needs are likely to be similar despite different underlying pathology, patients with atypical parkinsonian disorders are not excluded.

#### Exclusion criteria

Patients with PD in HY stages I-III are excluded as well as patients with a diagnosis of “symptomatic PD” such as normal pressure hydrocephalus or drug-induced Parkinsonism, except if persisting following discontinuation of the causative drug.

Assessments (see Table 1) are conducted in person at baseline and at 12 months, with optional telephone follow-up at 6 and 18 months.

**Table 1:** Assessment instruments and time points of application

Scales/Domains	Instruments	Reference	Application at			
			T1	T2	T3	T4
Patient-completed						
Quality of life and health status	EuroQol instrument	[20]	x	x	(x)	(x)
	PDQ-8^a^	[21]	x	x	x	x
Meaning in Life	SMiLE	[22]	x	x	(x)	(x)
Satisfaction with Care	Likert scale		x	x	(x)	(x)
Carer-completed						
Quality of life and health status	EuroQol instrument	[20]	x	x	(x)	(x)
Patient Quality of Life	DEMQOL-Proxy^b^	[23]	x	x	x	x
Satisfaction with Support	Likert Scale		x	x	(x)	(x)
Caregiver burden	Zarit burden scale	[24]	x	x	(x)	(x)
Clinician-completed						
Activities of daily living	UPDRS (pt. II)	[25]	x	x	x	x
	Schwab&England	[16]	x	x	x	x
Demographic/social data			x			
Checklist of symptoms, treatments, tests						
Comorbidities	Charlson-Index	[34]	x	x	(x)	(x)
Resource Utilisation questionnaire		[19]	x	x	x	
Clinical rating/judgement	CGI	[26]	x	x	x	x
	Hoehn and Yahr	[8]	x	x	(x)	(x)
	UPDRS (pts. I, III, IV)	[25]	x	x	(x)	(x)
Cognitive assessment	MMSE+clock drawing+fluency	[27–29]	x	x	(x)	(x)
	Pill questionnaire	[18]	x	x	(x)	(x)
Neuropsychiatric and other non-motor symptoms	Neuropsychiatric Inventory (NPI-12)	[30]	x	x	(x)	(x)
	Non-motor symptom scale	[31]	x	x	x	x
	Geriatric Depression Scale (GDS)	[32]	x	x	(x)	(x)
Basic palliative assessment	ESAS-(PD)	[33]	x	x	(x)	(x)
Supine and standing blood pressure			x			
Evaluation of Implementation				x		

^a^in patients with a diagnosis Parkinson’s disease only; ^b^in patients with dementia instead of PDQ8 and EQ-5D; (×) optional; T1 = Baseline with randomisation, T2: 6 months after baseline (in-person for intervention participants only), T3: 12 months after baseline (telephone with patient and/or carer or optional in-person assessment), and T4: 18 months after baseline (telephone with patient and/or carer or optional in-person assessment). Abbreviations used: *PDQ-8* The Parkinson’s Disease Questionnaire, short form, *SMiLE* Schedule for Meaning in Life Evaluation. Euroqol-Instrument (EQ-5D Index and visual analogue scale), *DEMQOL-Proxy* health-related quality of life for people with dementia - proxy version. *CGI* Clinical Global Impression, *UPDRS* Unified Parkinson’s Disease Rating Scale, *UPDRS* Unified Parkinson’s Disease Rating Scale, *MMSE* Mini Mental State Examination, *ESAS-PD* Edmonton Symptom Assessment System – Parkinson’s Disease

### Interventional trial

#### Inclusion criteria

For the interventional trial, patients are eligible if they experience at least one of the following insufficiently treated symptoms/problems (based on clinical judgment): motor parkinsonism according to Unified Parkinson’s Disease Rating Scale (UPDRS; including nocturnal motor problems), levodopa-induced motor complications, including Off-time > 50% of waking day (assessed on UPDRS part IV), moderately disabling dyskinesias (item 33 ≥ 2) or Off time dystonia, PD dementia (defined according to MDS Task Force definition [18], depression (GDS > 4 points) not receiving adequate treatment, psychotic symptoms, agitation/ aggression; anxiety and irritability/ liability (all NPI items > 4 points), symptomatic orthostatic hypotension, pain, constipation, urinary symptoms, insomnia or daytime sleepiness, falls OR are treated medications potentially associated with exacerbation of PD-related problems: (a) typical antipsychotics other than quetiapine or clozapine, anticholinergics, benzodiazepines, pills with protein rich meal, antihypertensives in symptomatic hypotensive patients, valproate, calcium antagonists, other medications with side effect exacerbating PD motor or non-motor symptoms OR are at risk of contractures and skin ulceration OR inadequate management of dysphagia with risk of choking, of dysarthria or of hypersalivation OR live in an inadequate home environment.

#### Exclusion criteria

Patients with parkinsonism seen by a movement disorders specialist in the four months prior to inclusion, and patients unable to comply with management plans (such as attending physiotherapy or change of medication) are excluded from the intervention trial.

Participants fulfilling the inclusion criteria are randomised in an open-label trial to two-arms in 3:1 allocation: to an intervention or care as usual, and assessed at 6 months (in-person; primary outcome) and optionally at 12 and 18 months (telephone review, secondary outcome). The intervention consists of individually tailored treatment strategies suggested by specialists after review of the baseline assessment and treatment regime.

### Qualitative study

#### Inclusion criteria

For the qualitative study, approximately 10 PD patients and an informal carer in each participating center (London, UK, Lund, Sweden, and Lisbon, Portugal) are being recruited consecutively. For inclusion, the same criteria apply as for the cohort study.

#### Exclusion criteria

In addition to the exclusion criteria of the cohort study, patients unable to communicate in words (because of either dysarthria or language problems) are excluded from the qualitative study.

Semi-structured interviews with patients and informal carers are conducted on the needs of patients with late stage Parkinsonism. The questions addressed are: perceived met and unmet needs, including palliative care needs, the experience and perceived impact of receipt of services, formal and informal support, exploration of deficits and barriers to adequate care provision, identification of factors influencing the decision as to whether patients are cared for at home or in residential care/institution, and advanced care planning (attitudes of patients towards advance directives and preferred place of death). These interviews are audio-recorded and transcribed; and then subject to thematic analysis aided by the N-VIVO computer program to identify the range of experiences and perceived needs and outcomes of different services provisions and treatments.

### Recruitment strategies

The major challenge in the CLaSP project is the identification, recruitment, and assessment of patients in late stages. We particularly aim to include patients who are not under regular specialist follow-up. Therefore, several methods to reach this target group are employed, particularly aiming to recruit individuals not currently attending routine specialist clinics: The centers contacted general practitioners, hospitals, nursing homes, patient advocate groups as well as self-help groups to draw attention to the CLaSP project and identify and recruit eligible patients.

### Clinical assessment

#### Cohort study

The assessment of patients and carer comprise of standardised questionnaires to evaluate disease severity, comorbidities, depression, cognition, non-motor symptoms, quality of life in patients and carers as well as caregiver burden (for an overview of timepoints and instruments/ questionnaires applied, see Table 1). The patients of the cohort study are followed up in person at 12 months and optionally via telephone at 6-month and 18 months. A special resource use questionnaire for patients with Parkinson’s disease and their carers was developed and used in a previous health economic cost of illness study [19] was applied. The questionnaire was adapted to the requirements of the respective country-specific health care system.

#### Intervention study

Baseline assessments are repeated at 6 months (T2) in person and on the telephone at 12 months. In addition, at T2, information is collected whether the individual intervention has been implemented into the patient’s treatment schedule.

### Outcome measures

The following instruments are used to collect data on the patients and their caregivers at different time points during the study (see also Table 1):

Health-related quality of life in PD patients and their caregivers is evaluated using the self-completed, generic EuroQol instrument, which comprises a questionnaire (EQ-5D) and a visual analogue scale (EQ VAS) [20]. The questionnaire consists of five questions with three levels of possible answers, representing the dimensions Mobility, Self-Care, Usual Activities, Pain/Discomfort, and Anxiety/Depression. In the EQ VAS, the participants rate their subjective health status on a scale with a range of 0 to 100 with higher scores indicating a better quality of life. Additionally, the self-administered, disease-specific PDQ-8 instrument is used in patients [21]. It is derived from the PDQ-39 and assesses eight domains: Activities of Daily Living, Attention and Working Memory, Communication, Depression, Quality of Life, and Social Relationships. Higher scores in the PDQ-8 indicate more problems and a worse quality of life. Satisfaction with Care is assessed in the patient via a 5-point Likert scale, with higher rating reflecting less satisfaction. The Schedule for Meaning in Life Evaluation (SMiLE) is an instrument to assess individual meaning in life [22]. First, the patients list one to seven areas that provide meaning to their lives and subsequently rate the current level of importance and satisfaction of each area. From these answers, a sum score can be calculated where higher scores indicate higher satisfaction in life. The DEMQOL proxy is used to obtain caregiver reports on the patient’s quality of life [23]. In our study, it is applied in patients with dementia instead of the PDQ-8 and the Euroqol instrument. Satisfaction with Support is assessed in the caregiver via a 5-point Likert scale, with higher rating indicating less satisfaction. The caregiver burden is assessed via the revised 22-items version of the Zarit Burden Scale [24]. Each item on the interview is answered by the caregiver on a 5-point scale with higher sum scores reflecting higher burden on the caregiver. The clinician completes the following assessments on the patient: For the clinical evaluation of Parkinson’s disease, the Unified Parkinson’s disease Rating Scale (UPDRS) is used to assess patients in four sections: Mentation, Behavior and Mood, Activities of Daily Living, Motor Examination, and Complications of Therapy [25]. In addition, the Hoehn and Yahr scale (HY) is used to describe the stage of PD severity [8]. The patient’s ability to perform activities of daily living is assessed with the Schwab & England Scale [16]. The score reflects the patient’s situation on a 0 (= complete dependence/bedridden) to 100% (= complete independence) scale. The Clinical Global Impression (CGI) rating scale is used to evaluate the patients’ symptom severity and change of symptoms over time [26]. The Mini-Mental State Examination (MMSE) is an assessment tool for general cognitive impairment, with higher overall total scores (range 0–30) indicating better performance [27]. The clock-drawing test is used for screening for cognitive impairment and dementia [28], which is sensitive to visuo-spatial impairment. The verbal fluency test (letter s) is used as a short test of executive function [29]. The Pill Questionnaire is a screening tool for mild cognitive impairment in nondemented Parkinson’s disease patients, using the ability to remember Parkinson’s disease-specific medications as an indicator of cognitive function [18]. The Neuropsychiatric Inventory (NPI-12) is an informant-based instrument to assess the presence and severity of twelve neuropsychiatric symptoms (i.e. delusions, hallucinations, agitation/aggression, dysphoria/depression, anxiety, euphoria/elation, apathy/indifference, disinhibition, irritability/lability, aberrant motor behaviors, night-time behavioral disturbances, appetite/eating disturbances) in patients with dementia, as well as informant distress [30]. To assess the occurrence and severity of non-motor symptoms, the Non-Motor Symptom Scale (NMSS) is used [31]. The Geriatric Depression Scale (GDS) is a 15-item screening tool with higher overall total scores (range 0–15) indicating higher depression levels [32]. The modified Edmonton Symptom Assessment System Scale for PD (ESAS-PD) was modified from symptom assessment in palliative care for patients with PD [33]. Comorbidities are assessed with the Charlson Comorbidity Index (CCI) [34].

#### Primary endpoint

The primary endpoint is the absolute change in UPDRS-ADL score from baseline to month 6 (intervention study) and to month 12 (cohort study). The UPDRS is administered by a researcher blinded to the treatment group.

#### Secondary endpoints

Secondary endpoints are the patients’ quality of life, mental health, disease severity and disability, non-motor symptoms scale score, occurrence of disease severity milestones (psychosis, dementia, falls, wheelchair-bound, institutionalization, and death), satisfaction with care as well as caregiver burden.

### Ethical approval

The CLaSP study is being conducted in compliance with the Helsinki Declaration [35], i.e. detailed oral and written information is given to the patients and their informant to ensure that the patient fully understands potential risks and benefits of the study. The study protocol was approved by the local ethics committees of all participating study sites (London:Camden and Islington NRES Committee 14/LO/0612, Bordeaux: South West and Overseas Protection Committee III (South West and Overseas Protection Committee). 2014-A01501–46, Lisbon:Centro Hospitalar Lisboa Norte, DIRCLN-19SET2014–275, Lund: EPN Regionala etikprovningsnamnden: Lund (EPN Regional Ethics Name: Lund). *JPND NC 559–002,* Marburg: Ethik-Kommission bei der Landesarztekammer Hessen (Ethics Commission at the State Medical Association Hesse). MC 309/2014, Munich: Ethikkommission bei der LMU Munchen (Ethics committee at the LMU Munchen). 193–14, Nijmegen: Radboud universitair medisch centrum, Concernstaf Kwaliteit en Veiligheid, Commissie Mensgebonden Onderzoek Regio Arnhem-Nijmegen (Radboud university medical center,Group staff Quality and Safety Human Research Committee, Arnhem-Nijmegen region). DJ/CMO300).

### Informed consent

Participants (patients and their caregivers) are included in the study after giving their written informed consent. In case the patient lacks capacity to give consent to the study due to severe cognitive impairment, the decision on study participation is made by a legal guardian or consultee, depending on the ethical and legal requirements at each site. All participants (patients and caregivers) can withdraw from the study at any point in time without any negative implications.

### Sample size

For the baseline evaluation of the **Cohort study**, at least 70 patients will be recruited per country. Out of this sample, 48 patients per country will be randomised for the intervention study. Permuted block randomization is used, stratified by country, dementia (yes/ no) and residence (nursing home or similar/ home). Applying a one-way ANOVA at a significance level of 5% and power of 80%, a sample size of 70 per country allows to detect differences in UPDRS-ADL scores between health care systems with a standard deviation of means of 1.76. The common standard deviation within each county is assumed to be 10.

For the **Intervention study**, the power calculations were based on the analysis of the primary efficacy endpoint: absolute change in UPDRS-ADL (Unified Parkinson’s Disease Rating Scale – part II, activities of daily living) score from baseline to month 6 (time to complete the intervention). Assumptions for mean and standard deviation of change in UPDRS-ADL scores of the intervention group are based on results of a previous study using the UPDRS-ADL [36]. An independent sample *t*-test was used to determine the sample size needed for detecting a difference in change of 4.8 between the two treatment groups. Assuming a standard deviation of 10 for difference in change, a two-sided significance level of 5%, a power of 80%, and non-participating and dropout rates of 20% each, 216 patients were calculated to be needed for the intervention group and 72 patients to the standard group (3:1 allocation).

### Statistical analysis

Categorical variables will be analysed by absolute and relative frequency, continuous variables by median, mean, standard deviation, 95% confidence interval, minimum, and maximum. Differences in continuous variables between groups (e.g. countries) at time points will be tested by analysis of variance (ANOVA) and analysis of covariance (ANCOVA) or their nonparametric analogues, respectively. Differences between groups at time points assessed by proportions will be analysed by the Chi-square or Fisher’s exact test, if applicable, and logistic regression.

Changes in continuous variables between two time points will be evaluated by the paired t-test or Wilcoxon signed rank test, respectively. Changes in proportions between two time points will be analysed by the McNemar test. Longitudinal analyses will be performed by applying linear and generalized linear mixed models with patient or carer as random effect, main effects for country and time, as well as a country-by-time interaction term and possible confounders.

In the intervention study, all analyses of outcome parameters will be done in the intent-to-treat population. The primary efficacy analysis will be to investigate the treatment effect on absolute change in UPDRS-ADL score from baseline to month 6 in the intervention and standard care group. Data will be analyzed using ANCOVA with categorical factors (treatment, country, dementia, residence) and baseline UPDRS-ADL score as a covariate. The null hypothesis “no difference in the primary endpoint between the intervention group and standard care group” will be tested against the alternative hypothesis “difference in the primary endpoint between both groups”. The primary efficacy analysis will be repeated using the per-protocol population to confirm the overall study results. Safety data will be analyzed in the as-treated population.

All tests will be performed two-sided, *p* values < 0.05 will be considered statistically significant.

## Discussion

Despite a large variety of symptomatic and supportive treatment options, PD remains a progressive and ultimately very disabling disorder, for which as yet no disease-modifying drugs exist. With the increasing population age and rising prevalence of PD expected over the next decades there is a growing challenge in the appropriate care for patients who reach the late stages of this disorder [37]. Improvements in care of late stage Parkinsonism are likely not only to improve patients’ health-related quality of life and caregiver burden, but also to reduce health care costs substantially by reducing the rate of institutionalization, hospital admissions, and polypharmacy. The CLaSP study is the first study that specifically characterises the clinical features, comorbidities, health care and social care needs, current treatment strategies and outcomes of patients with late stage parkinsonism across several European countries. It will evaluate the impact on patients as well as their carers, identify the current provision of health care and how it meets these needs, evaluate the adequacy of standard assessment methods and examine whether specialised, tailored review improves outcomes in patients with parkinsonism. It will also provide essential information on health economic data on the costs of providing health and social care for patients with this condition. Combining the cohorts' detailed assessments, using quantitative and qualitative data, in six different countries and across neurology, geriatric and palliative care settings, and studying this cohort longitudinally, will provide multifaceted, in-depth knowledge on this little studied population. This information can then inform how best to provide effective and cost-effective health and social care for this severely affected patient group and contribute to improved practices for clinical care.

## Abbreviations

ANCOVA: Analysis of covariance
ANOVA: Analysis of variance
CLaSP: Care of Late-Stage Parkinsonism
GDS: Geriatric Depression Scale
HY: Hoehn and Yahr stage
MDS: Movement Disorder Society
NPI: Neuropsychiatric Inventory
PD: Parkinson’s disease
UPDRS-ADL: Unified Parkinson’s Disease Rating Scale – part II, Activities of daily living
